# Genome-Wide Investigation of G6PDH Gene in Strawberry: Evolution and Expression Analysis during Development and Stress

**DOI:** 10.3390/ijms23094728

**Published:** 2022-04-25

**Authors:** Diya Lei, Yuanxiu Lin, Mengwen Luo, Bing Zhao, Honglan Tang, Xuan Zhou, Wantian Yao, Yunting Zhang, Yan Wang, Mengyao Li, Qing Chen, Ya Luo, Xiaorong Wang, Haoru Tang, Yong Zhang

**Affiliations:** 1 College of Horticulture, Sichuan Agricultural University, Chengdu 611130, China; 20152539@stu.sicau.edu.cn (D.L.); linyx@sicau.edu.cn (Y.L.); rokki95@hotmail.com (M.L.); zhaobing199892@gmail.com (B.Z.); 2020305037@stu.sicau.edu.cn (H.T.); 2021205001@stu.sicau.edu.cn (X.Z.); 2021305082@stu.sicau.edu.cn (W.Y.); asyunting@gmail.com (Y.Z.); wangyanwxy@163.com (Y.W.); limy@sicau.edu.cn (M.L.); supnovel@gmail.com (Q.C.); luoya945@163.com (Y.L.); wangxr@sicau.edu.cn (X.W.); htang@sicau.edu.cn (H.T.); 2 Institute of Pomology & Olericulture, Sichuan Agricultural University, Chengdu 611130, China

**Keywords:** strawberry, G6PDH, genome-wide, expression patterns, abiotic stress

## Abstract

As one of the key enzymes in the pentose phosphate pathway (PPP), glucose-6-phosphate dehydrogenase (G6PDH) provides NADPH and plays an important role in plant development and stress responses. However, little information was available about the G6PDH genes in strawberry (*Fragaria* × *ananassa*). The recent release of the whole-genome sequence of strawberry allowed us to perform a genome-wide investigation into the organization and expression profiling of strawberry G6PDH genes. In the present study, 19 strawberry G6PDH genes (FaG6PDHs) were identified from the strawberry genome database. They were designated as FaG6PDH1 to FaG6PDH19, respectively, according to the conserved domain of each subfamily and multiple sequence alignment with Arabidopsis. According to their structural and phylogenetic features, the 19 FaG6PDHs were further classified into five types: Cy, P1, P1.1, P2 and PO. The number and location of exons and introns are similar, suggesting that genes of the same type are very similar and are alleles. A cis-element analysis inferred that FaG6PDHs possessed at least one stress-responsive cis-acting element. Expression profiles derived from transcriptome data analysis exhibited distinct expression patterns of FaG6PDHs genes in different developmental stages. Real-time quantitative PCR was used to detect the expression level of five types FaG6PDHs genes and demonstrated that the genes were expressed and responded to multiple abiotic stress and hormonal treatments.

## 1. Introduction

The pentose phosphate pathway (PPP) is a principal glycol-metabolism pathway that plays an important role in growth, development, and physiological stresses in plants. The PPP produces nicotinamide adenine dinucleotide phosphate (NADPH), which is used in processes that include carbon fixation, fatty acid synthesis and nitrogen assimilation. Glucose-6-phosphate dehydrogenase (G6PDH, EC1.1.1.49) is recognized as a key enzyme of the plant PPP pathway, regulates the first step of dehydrogenation, and controls the reaction rate of the entire pathway in plants [1].

The G6PDH gene family members have been investigated in many plants, such as *Arabidopsis thaliana*, *Hevea brasiliensis* and *Glycine max*. There are six G6PDH genes in Arabidopsis [2], four in rubber tree [3] and nine in soybean [4]. According to their subcellular locations, G6PDH molecular variants are divided into cytosolic (Cy) and plastidic (P) isoforms, while the P-G6PDHs amino acid sequence is about 60 amino acids longer than the Cy-G6PDHs. It is encoded by nuclear genes, and the physicochemical properties of isoelectric point and pH are very similar. According to its specific antibody, post-translational redox modification level and amino acid sequence, the P-G6PDHs can be divided into the following types: P1 type, mainly present in green tissues; P2 type, which is insensitive to NADPH compared to P1 type; PO type, which rarely encodes a protein but can bind to the P1 type to allow P1 to enter the peroxisome. Cy-G6PDHs usually exhibits lower sensitivity to reducing power, which is regulated by NADPH/NADP+ ratio [5] and is competitively inhibited by NADPH [6]. Cy-G6PDHs have no allosteric regulation properties in terms of molecules, but subject to phosphorylation or other complex regulation [7]. The P-G6PDHs seem to be insensitive to NADPH and is susceptible to redox regulation [8,9]. The release of G6PDH from thylakoids requires an alkaline environment, and light can regulate the enzyme by regulating the pH dependence of the enzyme [10]. Several studies have shown that Cy-G6PDHs and plastidic P-G6PDHs were regulated by different factors, suggesting that there may be different response patterns in Cy-G6PDHs and plastidic P-G6PDHs under different stresses [11].

G6PDH is not only a rate-limiting enzyme, but also plays a role in response to biotic and abiotic stresses [3,12,13]. The response of plant G6PDH has been examined under different types of environmental stresses including drought [14,15,16], salinity [17,18,19,20], heavy metals [21,22], heat [23] and low temperature [24,25]. For years, the studies of G6PDHs have been mainly focused on aspects of transcription and activity analysis under various stresses, stressing their roles in maintaining cell redox balance to enhance stress resistance in plants [26,27,28].

Although the biological functions of G6PDH in stress responses have been described in several model plants, little information is known about strawberry (*Fragaria* × *ananassa*). Strawberry is a model fruit crop in Rosaceae genomics research and also has high economic and nutritional value. The environmental stresses significantly affect the growth and development of the strawberry plant. The strawberry fruit can be injured under low temperature, and the low temperature can also cause significant yield loss in strawberry production. Additionally, drought stress can result in diminished growth and the plant is usually severely damaged by salt stress. Due to the importance of the G6PDH genes in various physiological programs, it would be of interest to conduct a systematic investigation of the G6PDH family in strawberry. Recent completion of the strawberry genome sequencing provided an opportunity to reveal the organization, expression and evolutionary traits of strawberry G6PDH gene family at the genome-wide level [29]. In the present study, we identified 19 strawberry G6PDH genes and classified them into five main groups. Fifteen G6PDH genes of Fragaria× ananassa comprising one plastidic and four cytosolic isoforms were identified and each type of FaG6PDHs were cloned for the first time. The comprehensive analysis including the exon-intron organization, motif compositions, cis-elements, phylogenetic relationships, synteny analysis, tissue-specific expression levels and dynamic expression patterns in response to different abiotic stresses were further investigated. The results provide useful information for further functional characterization of G6PDH gene family members in strawberry.

## 2. Results

### 2.1. Identification Andanalysis of G6PDH Genes in Strawberry

G6PDH isozymes have various biochemical properties in plants [30]. A genome-wide analysis was performed to search all FaG6PDH genes and there were nineteen genes identified, which were named FaG6PDH1 to FaG6PDH19 according to their distribution on chromosomes. In order to analyze the biochemical properties of HvG6PDH proteins, we predicted the isoelectric point (pI), molecular weight, and subcellular localization of each FaG6PDH protein. The sequence length ranged from 3020 bp to 6330 bp, with a large difference; FaG6PDH11 was the shortest, and FaG6PDH14 was the longest. The deduced protein sequence are mainly 513 to 713 amino acids in length, except for FaG6PDH17 which has only 256 amino acids.

**Figure 1:**
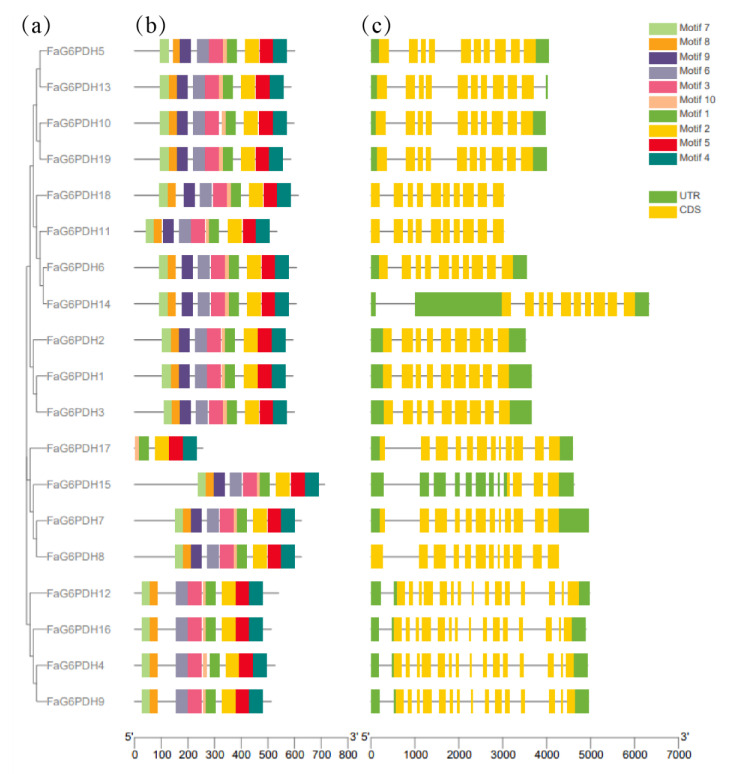
Gene structure and motif composition of FaG6PDH gene family. (**a**) Subgroup classification; (**b**) Conserved motif analysis; (**c**) Gene structural analysis.

The unrooted phylogenetic tree showed that the FaG6PDH genes were placed in five branches, which correspond to the evolution of different subgroups in *Arabidopsis thaliana*. Using MEME software to predict the full-length protein sequence (Figure 1), particularly FaG6PDH14 contains a long open reading frame (ORF). The number of exons in FaG6PDHs are mainly concentrated in 10–15, nearly all FaG6PDH genes have ten motifs for which the type and order are consistent, except for FaG6PDH17, which has only five motifs for which the type and order differ significantly from the others.,.

### 2.2. Characterization and Multiple Sequence Alignment of FaG6PDH Proteins

HMMER (v3.0) was used to identify the G6PDH gene family members from the *Fragaria × ananassa* genome based on the G6PDH domain sequence (Pfam: PF02781) and nineteen candidate G6PDH genes were found. The results analyzed by the ExPASy-ProtParam tool (Table 1) showed that the relative molecular mass of the nineteen FaG6PDH proteins ranged from 58.5 KDa to 70.8 KDa, except that FaG6PDH17 was only 29.1 KDa. The aliphatic index is expected to be between 84.88 and 92.10, and the more content of acidic amino acids in protein molecules with negatively GRAVY, indicating that FaG6PDH family proteins tend to be hydrophilic. These proteins do not have signal peptides or transmembrane structure, so they did not belong to membrane proteins or secreted proteins.

**Figure 2:**
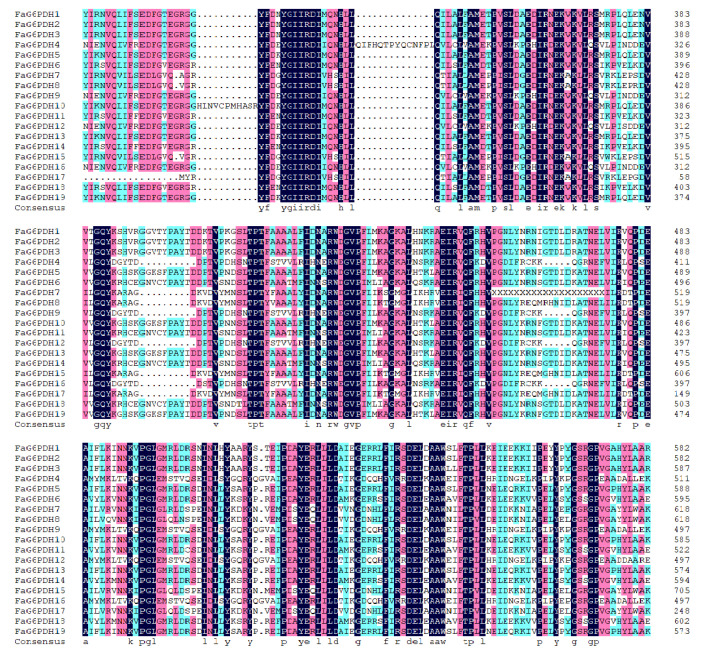
Multiple sequence alignment of the FaG6PDH domains.

In addition, multiple sequence alignments were performed on nineteen FaG6PDH proteins (Figure 2); the difference between the N-terminal sequences was larger, and FaG6PDH4, FaG6PDH9, FaG6PDH12 and FaG6PDH16 were significantly shorter than other amino acid sequences. In addition, FaG6PDHs contain the common Rossman fold, active site and NADP+ binding site of the G6PDH gene family and are relatively conservative.

**Figure 3:**
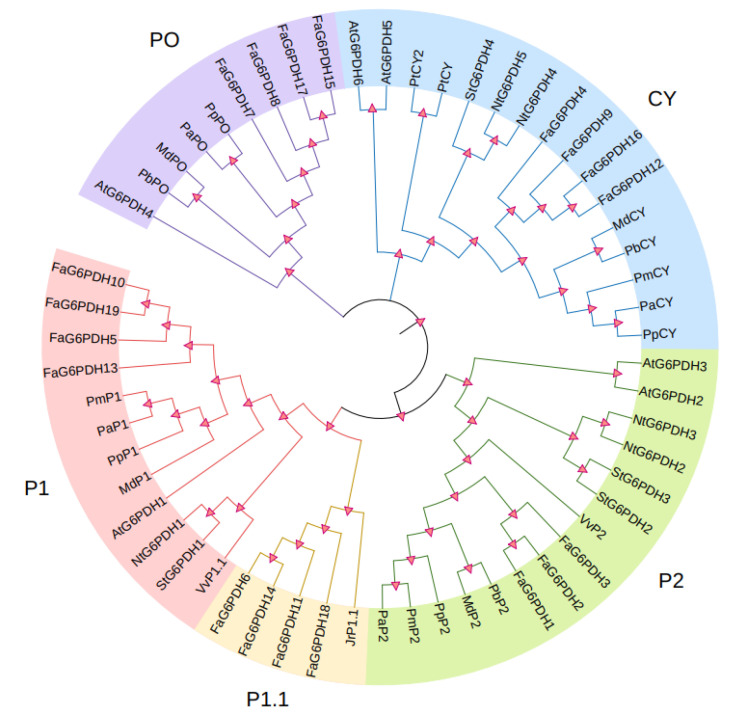
Phylogenetic analysis of FaG6PDHs. Five subgroups are shown as CY, P1, P1.1, P2, and PO according to the five known subgroups in Arabidopsis.

To explore the similarity between FaG6PDHs and other determined G6PDH proteins, using clustal X and MEGA, we constructed a phylogenetic tree based on a multiple alignment of FaG6PDHs and other 37 identified G6PDH proteins from *Arabidopsis thaliana*, tobacco, tomato, apple, pear, and grape etc. (Figure 3). It can be seen from the results that FaG6PDHs can be classified into five types: CY, P1, P1.1, P2, and PO. Based on the classification of G6PDH proteins in A. thaliana, FaG6PDH5, FaG6PDH10, FaG6PDH13 and FaG6PDH19 belong to P1 type; FaG6PDH6, FaG6PDH11, FaG6PDH14 and FaG6PDH18 belong to P1.1 type; FaG6PDH1–FaG6PDH3 belong to P2 type; FaG6PDH7, FaG6PDH8, FaG6PDH15 and FaG6PDH17 belong to PO type; and FaG6PDH4, FaG6PDH9, FaG6PDH12 and FaG6PDH16 belong to CY type. It is noteworthy that FaG6PDH has high homology with plants in Rosaceae and are far related to Arabidopsis, tobacco and other herbaceous plants.

### 2.3. Chromosomal Distribution and Synteny Analysis of FaG6PDH Genes

**Figure 4:**
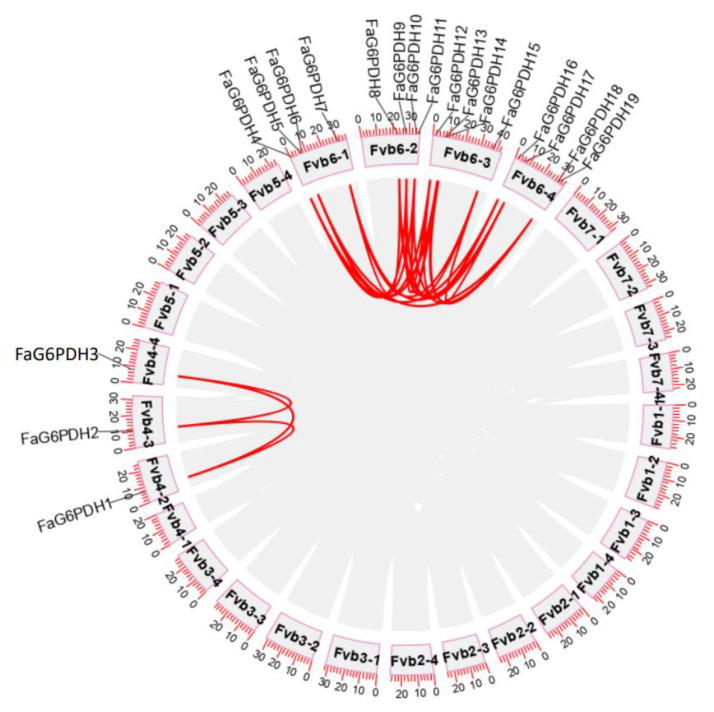
Synteny analysis of FaG6PDHs.

We choose the high-quality reference genome for the *Fragaria × ananassa* cultivar ‘Camarosa’, one of the most historically important and widely grown strawberry cultivars worldwide, to discuss FaG6PDH genes collinearity [31]. We investigated the chromosomal distribution of FaG6PDH genes in strawberry, all 19 genes were located on chromosomes (Figure 4). The nineteen FaG6PDH were mainly mapped on the LG6, and a few exist in LG4. Notably, chromosome Fvb4-1 does not contain any FaG6PDH family member.

### 2.4. Putative cis-Regulatory Elements in FaG6PDH Genes

**Figure 5:**
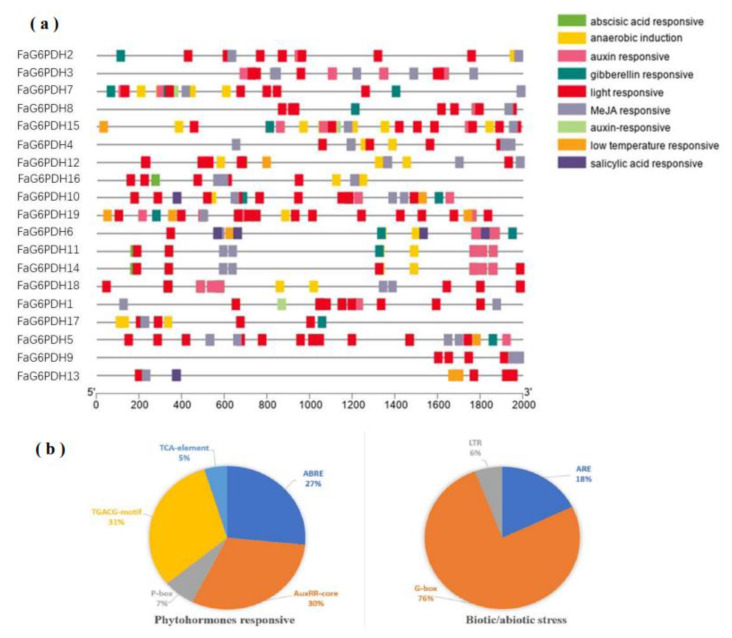
Investigation of cis-regulatory elements of FaG6PDH genes. They were categorized into two groups (phytohormones responsive, and biotic abiotic stress). (**a**) cis-acting elements of each category represented with different colors. (**b**) The size of the pie charts showed the percentage of promoter element in each category.

The cis-actng regulatory elements of each FaG6PDH gene were mainly divided into three physiological processes, including phytohormones, biotic/abiotic stress, and plant growth development responsiveness [32]. Most of the FaG6PDH genes contain TCA-elements: TGACG-motif, ABRE, ARE, and LTR (Figure 5). The phytohormone response-related cis-elements, such as ABRE (27%), AuxRR-core (30%), TCA-element (5%), P-box (7%) and TGACG-motif (31%) were also discovered, which are associated with ABA, auxin, SA, gibberellin and MeJA responses, indicating that these are involved in fruit ripening [33]. In addition, several kinds of stress-responsive elements in the abiotic/biotic stress related process were discovered, including LTR (6%), G-box (76%), and ARE (18%), which were related to cold, stress and light stress response, respectively. All FaG6PDHs contained G-box and TCA-elements, with high level TCA-elements copy numbers ranging from three (FaG6PDH13) to twelve (FaG6PDH5), which means the FaG6PDH family will be highly susceptible to light regulation. No other regulatory elements related to physiological processes were found in the promoter regions of FaG6PDH9 except G-box and TCA-elements.

### 2.5. Expression Patterns of FaG6PDHs Genes in Different Fruit Developmental Stages

**Figure 6:**
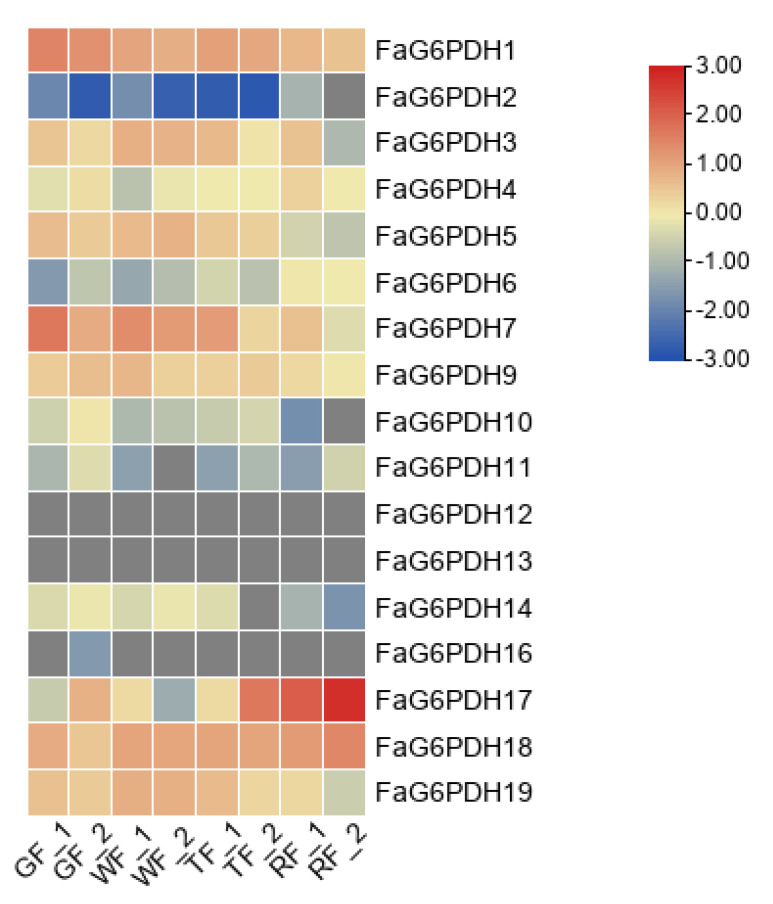
Heat map of FaG6PDH expression at different fruit development stages. qRTPCR data were normalized using Actin. GF: green fruit; WF: white fruit; TF: turning fruit; RF: red fruit.

We analyzed transcriptome data for expression of FaG6PDH genes in the green fruit, white fruit, turning fruit and red fruit. FaG6PDH12, FaG6PDH13 and FaG6PDH16 were barely expressed during the whole fruit development stage (Figure 6). In contrast, both FaG6PDH18 and FaG6PDH1 were highly expressed in the whole fruit development stage. The expression level of FaG6PDH17 was extremely high only at fruit ripening, which was similar to the expression pattern of FaG6PDH2. The expression patterns of the same subgroups were not similar, suggesting that there may be functional redundancy of genes in the subgroups.

### 2.6. Expression Profiles of FaG6PDHs under Different Abiotic Stresses

To further investigate the expression levels of G6PDH genes under various abiotic stresses, we selected five FaG6PDH genes according to the five known Arabidopsis species mentioned above (FaG6PDH−CY:FaG6PDH16; FaG6PDH-P1:FaG6PDH13; FaG6PDH-P1.1:FaG6PDH6; FaG6PDH-P2:FaG6PDH2; FaG6PDH-PO:FaG6PDH7) and analyzed their expression pattern using qRT-PCR after four treatments: drought, high salt, and high and low temperatures and spraying three types of hormones (SA, GA, and ABA) on fruits, and measured expression at six-hour intervals.

**Figure 7:**
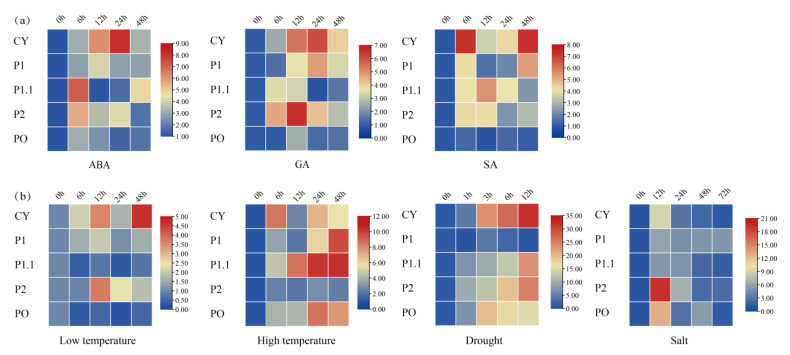
Expression pattern of FaG6PDH in fruit of strawberry under (**a**) hormones treatments and (**b**) stress treatments for 0 h to 72 h.

ABA usually mediates plant responses to abiotic stress. The qRT-PCR results showed the following under the treatment of abscisic acid (Figure 7): all of the expression of FaG6PDH gene family increased at 6 h, FaG6PDH-CY was in the ascending phase in the first 24 h and then it decreased rapidly; the trend of FaG6PDH-P1.1 reached the highest value at 6 h, then decreased rapidly, and then showed an upward trend after 24 h treatment; FaG6PDH-PO gradually accumulated at the first 6 h, and then remained relatively stable. Thus, this indicates that the FaG6PDH gene family can be affected by ABA, but plastidic G6PDH have a different response time to cytosolic G6PDH. In the treatment of gibberellin, the changes in the FaG6PDH gene subgroups were almost the same. The expression of FaG6PDH-CY and FaG6PDH-P1 peaked at 24 h after treatment, while the highest expression of FaG6PDH-P2 and FaG6PDH-PO appeared at 12 h; FaG6PDH-P1.1 accumulated at 6 h after treatment, and then gradually reduced the expression. The expression of FaG6PDH-CY and FaG6PDH-P1 were still the same when treated with salicylic acid. The expression of FaG6PDH-CY and FaG6PDH-P1 was the same at 6 h, then the expression rapidly decreased, and then it began to increase after 12 h. FaG6PDH-P1.1 was expressed in the first 12 h. The amount gradually increased and reached the maximum value, then began to decrease; FaG6PDH-P2 also had the maximum expression at 6 h, then slowly decreased until 24 h, and then slowly increased. In general, these results suggested that hormones treatments have little effect on FaG6PDH-PO expression levels in strawberry, and the cytosolic G6PDH is more responsive than plastidic G6PDH in hormones treatments.

Furthermore, we examined and analyzed the response of FaG6PDH genes under high temperature, the expression patterns of FaG6PDH-CY and FaG6PDH-PO were similar, and the expression was highest at 6 h, then gradually decreased. The expression of FaG6PDH-P1, FaG6PDH-CY, and FaG6PDH-P2 in the first 12 h rose similarly after 12 h treatment; the expression level of FaG6PDH-PCY gene reached the peak at 48 h; FaG6PDH-PO and FaG6PDH-P1.1 barely expressed. At low temperature, the expression of FaG6PDH-CY increased first, then decreased and then increased, and the expression was the highest at 48 h. The expression of FaG6PDH-P2 did not change significantly within 6 h of treatment, then the expression level began to increase gradually, and then decreased gradually after 12 h of treatment. The expression of FaG6PDH-P1 increased slightly and FaG6PDH-P1.1 and FaG6PDH-PO decreased slightly. Under drought stress, the expression levels of FaG6PDH-CY, FaG6PDH-P1.1 and FaG6PDH-P2 were always increased, and the expression of FaG6PDH-CY was most obvious at 1–3 h. The expression levels of FaG6PDH-P1.1 and FaG6PDH-P2 accumulated rapidly within 1 h before drought; the expression of FaG6PDH-PO was also increased in the first 3 h, but then decreased. FaG6PDH-P1 was rarely affected by drought, and the expression level was also not changed significantly. Under salt stress, the expression patterns of FaG6PDH-PO were different with trend under drought stress, at 12 h, the peak of gene expression was observed, and then continuously decreased. The expression of FaG6PDH-CY and FaG6PDH-P2 decreased rapidly after 24 h, then decreased slowly, and FaG6PDH-PO increased slightly after the expression decreased rapidly to 24 h. In general, the cytosolic subgroup was significantly regulated by abiotic stress, and its expression level was significantly increased under drought and significantly decreased under salt stress, suggesting that the cytosolic G6PDH participated in different regulatory functions under different stresses.

## 3. Discussion

Precise and complete identification is vital for studying the function and evolution of a gene family. To date, the G6PDH gene family has been reported in many plants, including barley [34], wheat [35], Nicotiana tabacum [23], and sugarcane [12], but it has not been identified in strawberry, especially octoploid strawberry (*Fragaria × ananassa*). In our study, nineteen G6PDH genes were found in the strawberry genome using six G6PDHs that have been found in Arabidopsis [27,36], all of which are distributed in chromosome 6. From the phylogenetic tree, we found that the nineteen genes can be divided into five types, and the same type of gene is very similar in the number and position of exons, or type and order of motifs, suggesting that the same type of genes are alleles. The similarity rate between the same plants is high, indicating that G6PDH is highly conserved during evolution and is important for plant growth and development. However, the number of G6PDH isozymes and the number of each type are different in different plants. Different species have different range in each type, such as six G6PDHs in Arabidopsis, two in CY type, two in P2 type, nineteen G6PDHs in strawberry, only four in CY type, and eight in P1 type. G6PDH protein sequence in strawberry have Rossman folds, active sites and NADP+ binding sites, which is consistent with the results of Arabidopsis [36], barley [34], and plum [18]. These genes encode protein between 58.5 and 70.8 kDa, which are similar to other species; however, there are still several amino acid differences, suggesting that there may be a difference between the effects of G6PDH isoenzymes. In addition, as they are clustered in the same group, those homologs may be involved in similar functions.

The cytoplasmic type of G6PDH content is higher than the plastid type has been found in previous studies [28], and the expression of FaG6PDH-CY in strawberry was also found to be much higher than that of plastid type G6PDH. The expression level of FaG6PDH-CY was higher than that of plastid type in different developmental stages of fruit; the expression level of FaG6PDH-P2 gene remained at a low level from the small green stage to the white ripe stage, and rose sharply during the turning red stage, indicating that FaG6PDH-P2 may be involved in fruit coloration, which is similar to the results of Kong [12].

More studies have focused on the relationship between G6PDH expression patterns and stress. The results of q-PCR showed that different genes of FaG6PDHs had different expression patterns under different abiotic stresses, which may be different response pathways in different stresses. In addition to the change in the expression of PO type in GA and SA treatment, other genes were up-regulated, indicating that the PO type is not sensitive to these two hormones, and does not participate in their regulatory pathways, while other genes are positively regulated. In the study of Subban [37] and others, G6PDH activity was also found to be affected by SA and GA. Under high temperature stress, P2 type is not sensitive to it, and CY type rises rapidly in a short time. It may be involved in anti-high temperature related pathways, and the expression of other genes were also increased, which may be regulated by high temperature stress although non-significant. In the low temperature treatment, two genes and PO types of P1 type were not involved in stress regulation, and the first expression level of P2 type increased, which may play a role in the downstream of cold stress regulation. CY type is more sensitive to cold stress, can be quickly perceived and participate in regulation. Under salt stress, P1 type did not participate in regulation, and other genes expressed similar patterns, indicating that they may participate in the same regulatory pathway. Under drought stress, the P1 type did not participate in the anti-reverse pathway, the other gene expression levels increased which the patterns were similar, presumably playing a role in the same pathway. In soybean [26], tomato [38] and other plants, it was found that the trend of FaG6PDH response stress was consistent with the ABA trend, and it was speculated that FaG6PDH responded to stress through ABA pathway.

The expression levels of all genes in ABA treatment increased to varying degrees, indicating that the FaG6PDH gene family is affected by ABA. Wang [26] believes that stress induces ABA accumulation, which in turn activates NADPH oxidase to enhance H_2_O_2_ production. H_2_O_2_ acts as a signaling molecule and activates G6PDH activity to participate in plant resistance. Yang [39] found that G6PDH of CY type in Arabidopsis was closely related to ABA. The results of this experiment are consistent with previous studies, which prove that G6PDHs in strawberry can respond to ABA, and ABA as a signaling molecule participates in many stress processes, which may be one of the mechanisms of FaG6PDHs participating in stress. However, further trials are needed on how specific FaG6PDHs respond to stress.

In summary, although not all genes in the family respond to abiotic stress and the expression patterns are not identical, FaG6PDHs play an important role in stress and may involve cross-regulation of multiple signaling pathways. Therefore, the next step can further explore how FaG6PDH operates in the signal network, thus providing a basis for improving the stress resistance of strawberry.

## 4. Materials and Methods

### 4.1. Plant Materials and Treatments

*Fragaria × ananassa* cv. Benihoppe, ‘Chengdu, China’, a typical cultivated variety, was used throughout the study. The different tissues (roots, stems, young leaves, old leaves, flowers, fruits) at different developmental stages (small green, big green, light green, white ripe, turning red, full red) were collected separately for RNA extraction and used for further qRT-PCR analysis. To investigate the expression pattern in response to various stress and hormonal treatments, five types of FaG6PDH genes were selected for further qRT-PCR analysis. For phytohormone analysis, the plants were sprayed evenly on the front and back of the blade with 2 L of 20 mg/L GA, 50 μM ABA and 1 mM SA solution for 0, 6, 12, 24 and 48 h. For heat and cold stress treatments, the plantlets were subjected to 38 and 4 °C, respectively. The leaves were collected at 0, 6, 12, 24, 48 h in cold and heat treatment. For salinity treatments, the plants were incubated in the 100 mM NaCl solution for 0, 12, 24, 48, 72 h. For drought treatment, the seedlings were placed on dry filter papers above a work bench and dried at 25 °C for 0, 1, 3, 6 and 12 h. All treated tissue samples were immediately frozen in liquid nitrogen and stored at −80 °C for subsequent analysis.

### 4.2. Identification and Classification of the G6PDH Genes in Strawberry

The protein sequence of Arabidopsis G6PDH from the genome (TAIR10) downloaded from The Arabidopsis Information Resource (TAIR) (https://www.rabidopsis.org/) (accessed on 12 September 2021) was used as query to search against the genome database for strawberry (Fragaria_ananassa_v1.0.a2) (GDR, https://www.rosaceae.org) (accessed on 13 September 2021). The sequence with high score in the results was retrieved as potential G6PDH paralogous in cultivated strawberry (FaG6PDH). The number of amino acids, open reading frame (ORF) length, putative protein molecular weight (MW), and isoelectric point (pI) for each sequence were obtained using ExPASy ProtParam tool (http://web.expasy.org/protparam/) (accessed on 15 September 2021). To validate the search results, all candidate sequences were examined and analyzed by a simple modular architecture research tool (SMART) (http://smart.embl.de/) (accessed on 17 September 2021) and the Conserved Domain Database (CDD) (http://www.ncbi.nlm.nih.gov/Structure/cdd/wrpsb.cgi) (accessed on 19 September 2021). A Maximum Likelihood phylogenetic tree was generated and Poisson model with clustal X and MEGA.

### 4.3. Gene Structure and Conserved Motif Analysis of the FaG6PDH Genes

The exon-intron organization of FaG6PDH genes were determined by comparing predicted coding sequences with their corresponding full-length sequences using the software TBtools (https://github.com/CJ-Chen/TBtools/releases) (accessed on 25 September 2021). The MEME online program (http://meme- suite.org/tools/meme) (accessed on 28 September 2021) for protein sequence analysis was used to identify conserved motifs in the identified FaG6PDH proteins. The upstream 2 Kb sequences of each FaG6PDH were retrieved from the corresponding gene as the putative promoter regions. The distribution of cis-acting regulatory elements in the promoter regions were identified using PlantCARE online software (http://bioinformatics.psb.ugent.be/webtools/plantcare/html/) (accessed on 30 September 2021). Gene synteny analysis was also conducted by TBtools software.

### 4.4. Expression Analysis of FaG6PDH Genes in Strawberry

Total RNA of strawberry tissues, fruits and different abiotic stress-treated leaves was extracted by modified CTAB method. After electrophoresis and nucleic acid protein assay, synthesis of first strand of cDNA by using the reverse transcription kit (Bao Bio) instructions for subsequent PCR experiments. qPCR-based expression analyses were carried out using SYBR Green Premix Ex TaqTM (Takara, Japan) on a CFX96 qPCR system (Bio-Rad, Chengdu, China) in triplicate of each sample. The housekeeping FaACTIN gene was used as an internal control. Each reaction was performed in biological triplicates and the relative expression was calculated using the 2-ΔΔCt method. Sequences of the primers and results used in this study were shown in detail in Supplemental Tables S1 and S2. The RNAseq-based expression levels of FaG6PDH genes in strawberry were retrieved from the online transcriptomic data (SRA accession: SRX6381727).

## 5. Conclusions

In summary, although not all genes in the family respond to abiotic stress, and the expression patterns are not identical, FaG6PDHs play an important role in stress and may involve cross-regulation of multiple signaling pathways. Therefore, the next step can further explore how FaG6PDH operates in the signal network, thus providing a basis for improving the stress resistance of strawberry.

## Figures and Tables

**Table 1:** List of FaG6PDHs identified in strawberry.

Gene Name	Gene ID	Chromosome	Location	Length (bp)	Number of Amino Acid	Protein MW (KDa)	Protein pI	Protein GRAVY
FaG6PDH1	FxaC_15g15650	Chr4-2	7391825..7395478(+)	3653	594	66.9	8.43	−0.315
FaG6PDH2	FxaC_13g22470	Chr4-3	10631546..10635068(+)	3522	594	66.9	8.57	−0.32
FaG6PDH3	FxaC_14g15070	Chr4-4	7414087..7417728(+)	3641	599	67.4	8.43	−0.324
FaG6PDH4	FxaC_21g03340	Chr6-1	1504970..1509902(+)	4932	527	60.2	6.07	−0.209
FaG6PDH5	FxaC_21g16460	Chr6-1	7345730..7349767(−)	4037	601	68.4	7.62	−0.411
FaG6PDH6	FxaC_21g16710	Chr6-1	7448648..7452176(−)	3528	607	69.4	5.96	−0.368
FaG6PDH7	FxaC_21g61380	Chr6-1	31504410..31509352(+)	4942	626	66.7	7.25	−0.291
FaG6PDH8	FxaC_23g35580	Chr6-2	22990853..22995565(+)	4712	626	70.8	5.94	−0.332
FaG6PDH9	FxaC_23g45900	Chr6-2	28274042..28278994(+)	4952	513	58.6	6.02	−0.224
FaG6PDH10	FxaC_23g59070	Chr6-2	34630394..34634363(−)	3969	598	68	7.64	−0.41
FaG6PDH11	FxaC_23g59250	Chr6-2	34709249..34712269(−)	3020	534	61.2	5.44	−0.378
FaG6PDH12	FxaC_22g02420	Chr6-3	1244434..1249416(+)	4982	540	61.6	5.89	−0.165
FaG6PDH13	FxaC_22g14340	Chr6-3	6979140..6983155(−)	4015	587	66.7	6.75	−0.369
FaG6PDH14	FxaC_22g14700	Chr6-3	7086714..7093044(−)	6330	606	69.3	6.31	−0.318
FaG6PDH15	FxaC_22g60560	Chr6-3	37112791..37117415(+)	4624	713	80.6	5.96	−0.345
FaG6PDH16	FxaC_24g02960	Chr6-4	1307649..1312541(−)	4892	513	58.5	5.76	−0.222
FaG6PDH17	FxaC_24g12040	Chr6-4	5813786..5818374(−)	4588	256	29.1	5.17	−0.315
FaG6PDH18	FxaC_24g49360	Chr6-4	29924067..29927094(+)	3027	614	70.3	6.07	−0.378
FaG6PDH19	FxaC_24g49610	Chr6-4	30017351..30021341(+)	3990	586	66.5	6.96	−0.418

## Supplementary Materials

The following supporting information can be downloaded at: https://www.mdpi.com/article/10.3390/ijms23094728/s1.

## References

[1] Esposito S., Massaro G., Vona V., Di Martino Rigano V., Carfagna S. (2003). Glutamate synthesis in barley roots: The role of the plastidial glucose-6-phosphate dehydrogenase. Planta.

[2] Wakao S., Benning C. (2005). Genome-wide analysis of glucose-6-phosphate dehydrogenases in Arabidopsis. Plant J..

[3] Long X., He B., Fang Y., Tang C. (2016). Identification and characterization of the glucose-6-phosphate dehydrogenase gene family in the para rubber tree, Hevea brasiliensis. Front. Plant Sci..

[4] Zhao Y., Cui Y., Huang S., Yu J., Wang X., Xin D., Chen Q. (2020). Genome-wide analysis of the glucose-6-phosphate dehydrogenase family in soybean and functional identification of GmG6PDH2 involvement in salt stress. Front. Plant Sci..

[5] Castiglia D., Cardi M., Landi S., Cafasso D., Esposito S. (2015). Expression and characterization of a cytosolic glucose 6 phosphate dehydrogenase isoform from barley (*Hordeum vulgare*) roots. Protein Expr. Purif..

[6] Jain M., Brenner D.A., Cui L., Lim C.C., Wang B., Pimentel D.R., Liao R. (2003). Glucose-6-phosphate dehydrogenase modulates cytosolic redox status and contractile phenotype in adult cardiomyocytes. Circ. Res..

[7] Kotaka M., Gover S., Vandeputte-Rutten L., Au S.W., Lam V.M., Adams M.J. (2005). Structural studies of glucose-6-phosphate and NADP+ binding to human glucose-6-phosphate dehydrogenase. Acta Crystallogr. Sect. D Biol. Crystallogr..

[8] Dal Santo S., Stampfl H., Krasensky J., Kempa S., Gibon Y., Petutschnig E., Jonak C. (2012). Stress-induced GSK3 regulates the redox stress response by phosphorylating glucose-6-phosphate dehydrogenase in Arabidopsis. Plant Cell.

[9] Née G., Aumont-Nicaise M., Zaffagnini M., Nessler S., Valerio-Lepiniec M., Issakidis-Bourguet E. (2014). Redox regulation of chloroplastic G6PDH activity by thioredoxin occurs through structural changes modifying substrate accessibility and cofactor binding. Biochem. J..

[10] Ben B.D., Anderson L.E. (1981). Light-induced release of bound glucose-6-phosphate dehydrogenase to the stroma in pea chloroplasts. Plant Physiol..

[11] Yang L., Wang X., Chang N., Nan W., Wang S., Ruan M., Bi Y. (2019). Cytosolic glucose-6-phosphate dehydrogenase is involved in seed germination and root growth under salinity in Arabidopsis. Front. Plant Sci..

[12] Yang Y., Fu Z., Su Y., Zhang X., Li G., Guo J., Xu L. (2014). A cytosolic glucose-6-phosphate dehydrogenase gene, ScG6PDH, plays a positive role in response to various abiotic stresses in sugarcane. Sci. Rep..

[13] Sergio E. (2016). Nitrogen Assimilation, Abiotic Stress and Glucose 6-Phosphate Dehydrogenase: The Full Circle of Reductants. Plants.

[14] Landi S., De Lillo A., Nurcato R., Grillo S., Esposito S. (2017). In-field study on traditional Italian tomato landraces: The constitutive activation of the ROS scavenging machinery reduces effects of drought stress. Plant Physiol. Biochem..

[15] Liu Y., Yan F., Ding M., Li X., Wang L., Yue S., Liang W. (2017). Clone of G6PDH Gene(GND)from Nostoc flagelliforme and Differential Expression under Drought Stress. Acta Bot. Boreali-Occident. Sin..

[16] Liu J., Wang X., Hu Y., Hu W., Bi Y. (2013). Glucose-6-phosphate dehydrogenase plays a pivotal role in tolerance to drought stress in soybean roots. Plant Cell Rep..

[17] Valderrama R., Corpas F.J., Carreras A., GÓMEZ-RODRÍGUEZ M.V., Chaki M., Pedrajas J.R., Barroso J.B. (2006). The dehydrogenase-mediated recycling of NADPH is a key antioxidant system against salt-induced oxidative stress in olive plants. Plant Cell Environ..

[18] Wang X., Ma Y., Huang C., Wan Q., Li N., Bi Y. (2008). Glucose-6-phosphate dehydrogenase plays a central role in modulating reduced glutathione levels in reed callus under salt stress. Planta.

[19] Zhao C., Wang X., Wang X., Wu K., Li P., Chang N., Bi Y. (2015). Glucose-6-phosphate dehydrogenase and alternative oxidase are involved in the cross tolerance of highland barley to salt stress and UV-B radiation. J. Plant Physiol..

[20] Gao S., Zheng Z., Huan L., Wang G. (2016). G6PDH activity highlights the operation of the cyclic electron flow around PSI in Physcomitrella patens during salt stress. Sci. Rep..

[21] Wang S.H., Zhang H., He Q.Y. (2011). Effects of copper stress on antioxidant system in leaves of Alfalfa seedlings. J. Appl. Ecol..

[22] Wang H., Hou J., Li Y., Zhang Y., Huang J., Liang W. (2017). Nitric oxide-mediated cytosolic glucose-6-phosphate dehydrogenase is involved in aluminum toxicity of soybean under high aluminum concentration. Plant Soil.

[23] Gong H., Chen G., Li F., Wang X., Hu Y., Bi Y. (2012). Involvement of G6PDH in heat stress tolerance in the calli from Przewalskia tangutica and Nicotiana tabacum. Biol. Plant..

[24] Zhang Y., Luo M., Cheng L., Lin Y., Chen Q., Sun B., Tang H. (2020). Identification of the Cytosolic Glucose-6-Phosphate Dehydrogenase Gene from Strawberry Involved in Cold Stress Response. Int. J. Mol. Sci..

[25] Lin Y., Lin S., Guo H., Zhang Z., Chen X. (2013). Functional analysis of PsG6PDH, a cytosolic glucose-6-phosphate dehydrogenase gene from Populus suaveolens, and its contribution to low temperature tolerance improvement in tobacco plants. Biotechnol. Lett..

[26] Wang H., Yang L., Li Y., Hou J., Huang J., Liang W. (2016). Involvement of ABA- and H_2_O_2_-dependent cytosolic glucose-6-phosphate dehydrogenase in maintaining redox homeostasis in soybean roots under drought stress. Plant Physiol. Biochem..

[27] He Q., Li P., Zhang W., Bi Y. (2020). Cytoplasmic glucose-6-phosphate dehydrogenase plays an important role in the silicon-enhanced alkaline tolerance in highland barley. Funct. Plant Biol..

[28] Li C., Wei M., Ge Y., Zhao J., Chen Y., Hou J., Li J. (2020). The role of glucose-6-phosphate dehydrogenase in reactive oxygen species metabolism in apple exocarp induced by acibenzolar-S-methyl. Food Chem..

[29] Shulaev V., Sargent D.J., Crowhurst R.N., Mockler T.C., Folkerts O., Delcher A.L., Folta K.M. (2011). The genome of woodland strawberry (*Fragaria vesca*). Nat. Genet..

[30] Cardi M., Chibani K., Castiglia D., Cafasso D., Pizzo E., Rouhier N., Esposito S. (2013). Overexpression, purification and enzymatic characterization of a recombinant plastidial glucose-6-phosphate dehydrogenase from barley (*Hordeum vulgare* cv. Nure) roots. Plant Physiol. Biochem..

[31] Liston A., Wei N., Tennessen J.A., Li J., Dong M., Ashman T.L. (2020). Revisiting the origin of octoploid strawberry. Nat. Genet..

[32] Manzoor M.A., Li G., Abdullah M., Han W., Wenlong H., Yang Z., Cai Y. (2021). Genome-wide investigation and comparative analysis of MATE gene family in Rosaceae species and their regulatory role in abiotic stress responses in Chinese pear (*Pyrus bretschneideri*). Physiol. Plant..

[33] Zhang Y., Li Q., Xu L., Qiao X., Liu C., Zhang S. (2020). Comparative analysis of the P-type ATPase gene family in seven Rosaceae species and an expression analysis in pear (*Pyrus bretschneideri* Rehd.). Genomics.

[34] Feng R., Wang X., He L., Wang S., Li J., Jin J., Bi Y. (2020). Identification, Characterization, and Stress Responsiveness of Glucose-6-phosphate Dehydrogenase Genes in Highland Barley. Plants.

[35] Nemoto Y., Sasakuma T. (2000). Specific expression of glucose-6-phosphate dehydrogenase (G6PDH) gene by salt stress in wheat (*Triticum aestivum* L.). Plant Sci..

[36] Landi S., Capasso G., Esposito S. (2021). Different G6PDH isoforms show specific roles in acclimation to cold stress at various growth stages of barley (*Hordeum vulgare*) and Arabidopsis thaliana. Plant Physiol. Biochem..

[37] Subban K., Subramani R., Srinivasan V.P.M., Johnpaul M., Chelliah J. (2019). Salicylic acid as an effective elicitor for improved taxol production in endophytic fungus Pestalotiopsis microspora. PLoS ONE.

[38] Landi S., Nurcato R., De Lillo A., Lentini M., Grillo S., Esposito S. (2016). Glucose-6-phosphate dehydrogenase plays a central role in the response of tomato (*Solanum lycopersicum*) plants to short and long-term drought. Plant Physiol. Biochem. PPB.

[39] Yang L., Wang S., Sun L., Ruan M., Li S., He R., Bi Y. (2019). Involvement of G6PD5 in ABA response during seed germination and root growth in Arabidopsis. BMC Plant Biol..

