# Supplementary material for: Genome-Wide Investigation of G6PDH Gene in Strawberry: Evolution and Expression Analysis during Development and Stress
Source: Int J Mol Sci. 2022 Apr 25;23(9):4728. doi: 10.3390/ijms23094728 (PMC9104510; doi:10.3390/ijms23094728)
Supplement: Supplementary file 1 [file ijms-23-04728-s001.zip › ijms-1670884-supplementary.pdf]

Table S1. Primers for quantitative RT-PCR

Gene name	Forward primers (5′ to 3′)	Reverse primers (5′ to 3′)	Product size (bp)
qPCR-FaG6PDH-CY	TCCTCCATCAGTATATCCATCT	TACGAGTCCATCCACCAT	83
qPCR-FaG6PDH-P1	CTGGTCTGTATAGTTCTGA	ATATGTTCGGAGGTATCG	114
qPCR-FaG6PDH-P1.1	GGTATCCAAGAGAGTTCCCAGA	ACTCCGACAGGTCCACTACTAC	193
qPCR-FaG6PDH-P2	TCTATCTATCTATTCCTCCAA	GGCTTCTCAACAATAACT	103
qPCR-FaG6PDH-PO	GATCAGTACGGAAATTGGAGCCTAG	CCGCAGCAAAGTATGTGGGAGT	114
qPCR-FaACTIN	CCAAGGCTAATCGTGAGAAGA	GTCGAGCACAATACCAGTAG	128

Table S2. RT-PCR relative expression results for heatmap

Low temperature	CY	P1	P1.1	P2	PO
0h	1	1	1	1	1
6h	2.098221	1.578033	0.381432	0.95871	0.331594
12h	3.578033	1.986685	0.760489	3.711569	0.546831
24h	1.635613	1.090896	0.301452	2.40605	0.220931
48h	4.672256	1.578741	0.725728	1.820288	0.511096
High temperature	CY	P1	P1.1	P2	PO
0h	1	1	1	1	1
6h	8.515858	3.532212	4.580712	2.471118	4.013872
12h	2.566852	2.148743	8.607367	2.148943	4.076978
24h	6.928293	6.135743	10.2491	2.825169	8.574188
48h	5.666665	9.644355	10.367	2.393374	7.709657
Drought	CY	P1	P1.1	P2	PO
0h	1	1	1	1	1
1h	4.800995	0.509034	7.382793	9.624200289	7.147583
3h	23.42537	4.518897	9.764188	12.29711364	19.70969
6h	26.78463	2.472265	12.79493	19578048064	17.20694
12h	32	0.729932	23.33618	24.94848793	15.11071
Salt	CY	P1	P1.1	P2	PO

0h	1	1	1	1	1
12h	9.063071	5.907305	5.184363	19.29293	12.79493
24h	3.08086	4.74311	5.005381	6.634556	2.876205
48h	2.163449	5.118898	2.246221	2.514027	5.404521
72h	1.313422	5.107085	1.61049	2.596677	1.389116

ABA	CY	P1	P1.1	P2	PO
0h	1	1	1	1	1
6h	3.111262	2.857988	7.146763	5.61778	3.111262
12h	6.206149	4.062872	1.084853	3.470547	2.483716
24h	8.073382	2.804027	1.607702	4.272262	1.433955
48h	3.254645	2.857988	4.773343	1.747146	1.774608

GA	CY	P1	P1.1	P2	PO
0h	1	1	1	1	1
6h	2.508527	1.398778	3.531536	4.648182	1.005212
12h	5.470838	3.649971	3.300078	6.568461	2.589188
24h	6.105037	4.821079	0.851159	4.250109	1.287882
48h	4.041958	3.386981	1.558329	2.938337	1.438934

SA	CY	P1	P1.1	P2	PO
0h	1	1	1	1	1
6h	7.235035	4.2	4.126232	4.295607	1.368409
12h	3.611254	1.577347	5.491538	4.198867	0.933033
24h	4.301978	2.066945	4.084049	2.321408	1.394744
48h	7.260153	5.32033	2.341609	3.116658	1.176907
